# Rare exonic *CELSR3* variants identified in Bladder Exstrophy Epispadias Complex

**DOI:** 10.3389/fgene.2024.1266210

**Published:** 2024-06-05

**Authors:** Angie C. Jelin, Nikolai Sopko, Nara Sobreira, Simeon A. Boyadjiev, Elizabeth Wohler, Christian Morrill, P. Dane Witmer, Jason Michaud, David Valle, John Gearhart, Heather Dicarlo

**Affiliations:** ^1^ Department of Gynecology and Obstetrics, Johns Hopkins School of Medicine, Baltimore, MD, United States; ^2^ Department of Genetic Medicine, Johns Hopkins School of Medicine, Baltimore, MD, United States; ^3^ Department of Pediatric Urology, Johns Hopkins School of Medicine, Baltimore, MD, United States; ^4^ Department of Pediatrics, Johns Hopkins School of Medicine, Baltimore, MD, United States; ^5^ Department of Pediatrics, University of California Davis, Davis, CA, United States

**Keywords:** bladder exstrophy, genetic variants, Celsr3, exome sequence, bladder growth

## Abstract

**Introduction/background:**

Bladder exstrophy epispadias complex (BEEC) is a rare congenital anomaly of unknown etiology, although, genetic and environmental factors have been associated with its development. Variants in several genes expressed in the urogenital pathway have been reported as causative for bladder exstrophy in human and murine models. The expansion of next-generation sequencing and molecular genomics has improved our ability to identify the underlying genetic causes of similarly complex diseases and could thus assist with the investigation of the molecular basis of BEEC.

**Objective:**

The objective was to identify the presence of rare heterozygous variants in genes previously implicated in bladder exstrophy and correlate them with the presence or absence of bladder regeneration in our study population.

**Patients and Methods:**

We present a case series of 12 patients with BEEC who had bladder biopsies performed by pediatric urology during bladder neck reconstruction or bladder augmentation. Cases were classified as “sufficient” or “insufficient” (n = 5 and 7, respectively) based on a bladder volume of greater than or less than 40% of expected bladder size. Control bladder tissue specimens were obtained from patients (n = 6) undergoing biopsies for conditions other than bladder exstrophy. Whole exome sequencing was performed on DNA isolated from the bladder specimens. Based on the hypothesis of *de novo* mutations, as well as the potential implications of autosomal dominant conditions with incomplete penetrance, each case was evaluated for autosomal dominant variants in a set of genes previously implicated in BEEC.

**Results:**

Our review of the literature identified 44 genes that have been implicated in human models of bladder exstrophy. Our whole exome sequencing data analysis identified rare variants in two of these genes among the cases classified as sufficient, and seven variants in five of these genes among the cases classified as insufficient.

**Conclusion:**

We identified rare variants in seven previously implicated genes in our BEEC specimens. Additional research is needed to further understand the cellular signaling underlying this potentially genetically heterogeneous embryological condition.

## Introduction

BEEC is a complex embryological defect of the abdominal wall that requires staged urological reconstructive surgeries (Stewart et al., 2015). BEEC has been proposed to be polygenic in the Comparative Toxicogenomics Database (CTD)-gene associations data set includes 4,457 genes/proteins associated with urogenital anomalies (Davis et al., 2015). In fact, 44 are implicated as causative for BEEC in either human or murine models, but their role in the disease mechanism is largely unknown (Stevenson, 2021).

Surgical planning and methodology for patients with BEEC is complex. Although bladder regeneration or expansion are critical predictors of outcome, they can only be measured over time (Bazinet et al., 2024). Patients with sufficient bladder size do not require augmentation cystoplasty later in life, as their bladders have grown with age to achieve adequate storage volumes (Khandge et al., 2023). In contrast, bladders of insufficient size demonstrate minimal growth with age and require augmentation cystoplasty later in life to achieve adequate storage volumes (Khandge et al., 2023). Identifying potential genotypic differences between these two clinical populations may aid in understanding factors important to bladder growth biology in the BEEC population and potentially assist in patient counseling of functional outcomes and future surgical requirements.

Whole exome sequencing (WES) has emerged as a cost-effective technique to identify single nucleotide variants in genes that encode proteins (Bekheirnia et al., 2016). This has allowed for significant advances in uncovering the genetic etiology of both single gene and complex diseases (Petrovski et al., 2019). WES can also be used to identify somatic mosaic variants in an affected tissue such as the bladder tissue from patients with bladder exstrophy.

We sought to evaluate bladder tissue from patients with bladder exstrophy, with and without sufficient bladder size, by whole exome sequencing.

Our objective was to identify the presence of rare heterozygous variants in genes previously implicated in BEEC and correlate them with the presence or absence of bladder regeneration in our study population.

## Materials and methods

### Bladder samples

Bladder tissue was obtained from patients with BEEC and controls who were undergoing care at a tertiary university-based setting. Patients who presented to pediatric urology for bladder neck reconstruction or bladder augmentation between July 2009 and July 2015 were consented for bladder biopsy. Full thickness bladder biopsies were obtained by a pediatric urologist in the operating room at the time of the procedure. Control samples were obtained from patients undergoing ureteral implantation for vesico-urinary reflux (VUR) with normal size and morphology of the urinary bladder. This study was approved by the Institutional Review Board of the Johns Hopkins University.

We queried medical records to determine the expansion of bladder growth over time in addition to patient demographics. We also obtained the initial bladder size and initial operative procedure. Patients presenting for a biopsy with expected bladder growth greater than 40% were classified as “sufficient” while those presenting with minimal growth, <40%, were classified as “insufficient”. All of our BEEC patients were non-syndromic, without associated anomalies or developmental delays. Additional patients undergoing ureteral implantation for VUR were also consented as controls.

### Whole exome sequencing

Whole exome sequencing was performed as previously described (Li et al., 2016) using the illumina HiSeq2500 Platform. Variants were analyzed utilizing the PhenoDB (Sobreira et al., 2015) tool to filter for rare, heterozygous variants with a minor allele frequency (MAF) < 0.01 in the 1,000 Genome Project, Exome Variant Server, gnomAD, and our collective in-house dataset (Sobreira et al., 2015). Following completion of the variant filtering pipeline, variants present in both BEEC cases and controls were excluded. Only variants meeting the following criteria were included: (1) filter = pass, (2) quality ≥ 100, (3) depth coverage ≥ 20X, and (4) variant fraction ≥ 20%. We utilized the integrated genomic viewer to ensure the variant read was supported. Variants identified in sufficient or insufficient cases from the curated list of 44 genes associated with BEEC in human models were selected for further analysis.

## Results

Patients with non-syndromic BEEC who underwent whole exome sequencing on DNA isolated from bladder biopsies included: five classified as sufficient, seven classified as insufficient, and six controls. Demographics of the three groups are provided in Table 1. The average percentage expected bladder volume of cases with sufficient bladder size was 67.7% and those with insufficient was 25.1%. In the majority of cases the initial procedure was performed in the neonatal period (8/12) (Table 2).

**TABLE 1:** Demographics of bladder exstrophy with sufficient or insufficient bladder volumes and controls.

Parameter		Sufficient (n = 5)	Insufficient (n = 7)	Control (n = 6)
Gender				
	Male	3	5	3
	Female	2	2	3
Race/Ethnicity				
	White	5	3	5
	African American	0	2	0
	Hispanic	0	1	1
	Arabian	0	1	0
Age at surgery	Ave (range)	9.0 (5.9–11.9)	9.8 (5.4–15.3)	6.7 (4.0–9.2)
% Expected Bladder Volume	Ave (range)	67.7 (43.3–92.8)	25.1 (23.2–28.5)	--

**TABLE 2:** Phenotypic information for BEEC with sufficient and insufficient growth.

Phenotype	Case	Initial procedure	Age of initial closure	Location of initial closure	Complete vs. staged	Osteotomy	Traction	Polypoid	Initial bladder size (age)	Procedure at time of biopsy
Sufficient	S1	Closure of Classic BE	Neonatal	JHH	Modern Staged	No	Yes	No	63 mL (10 months)	BNR
	S2	Closure of Classic BE	6 months	OSH	Complete Primary Repair	No	No	No	85 mL (3 years)	BNR
	S3	Closure of Classic BE	Neonatal	JHH	Complete Primary Repair	No	No	No	130 mL (4 years)	BNR
	S4	Closure of Female BE	Neonatal	JHH	Modern Staged	No	Yes	No	46 mL (6 months)	BNR
	S5	Closure of Female BE	Neonatal	JHH	Modern Staged	No	Yes	Yes	92 mL (2 years)	BNR
Insufficient	I1	Feminizing genitoplasty	1 year	JHH	Genitoplasty and urethroplasty	No	No	No	34 mL (1 year)	BA
	I2	Cloacal exstrophy repair	Neonatal	OSH	Single Stage Complete Repair	Yes	No	No	37 mL (3 years)	BA
	I3	Closure of Classic BE	Neonatal	OSH	Modern Staged	No	No	No	37 mL (5 years)	BA
	I4	Closure of Classic BE	2 years	OSH	NA	Yes	No	NA	70 mL (6 years)	BA
	I5	Closure of Classic BE	3 years	OSH	NA	No	NA	Yes	62 mL (3 years)	BA
	I6	Closure of Classic BE	Neonatal	OSH	Complete Primary Repair	No	Yes	No	50 mL (4 years)	BA
	I7	Closure of female BE	Neonatal	OSH	Modern Staged	NA	No	No	42 mL (3 years)	BA

Bladder exstrophy (BE), Johns Hopkins Hospital (JHH), Outside Hospital (OSH), Bladder neck reconstruction (BNR), Bladder Augmentation (BA), Not available (NA).

Our review of the literature identified 44 human genes implicated in the etiology of BEEC. We identified rare, candidate causative variants in seven genes on this list (*BMP10, WIZ, CYP4F22, CELSR1, CELSR3, TP63* and *WNT11*) in 2/5 cases classified as sufficient and in 5/7 cases classified as insufficient (Table 3). *CELSR3* variants were present in three cases with insufficient bladders; two in Caucasian patients, and one in a Hispanic patient. One of the patients with a variant in *CELSR3* also had variants in two other genes identified as associated with BEEC (*TP63* and *WNT11*). A *CELSR1* variant was identified in an African-American patient with insufficient bladder capacity. Variants in two cases with sufficient bladder capacity were identified in *BMP10* and *WIZ,* respectively (Table 3).

**TABLE 3:** Variants identified in genes previously implicated in urogenital anomalies.

Phenotype	Case	Gene	Transcript	Variant	OMIM conditions	ClinVar classification	Varsome classification	RVIS Intolerance Percentile	gnomAD (global)	CADD Score
Sufficient	S1	*BMP10*	NM_014482.2	c.599C>G (p.Thr200Ser)	---	---	Benign	0.09	0.0046	15.83
	S2	*WIZ*	NM_021241.2	c.1352A>G (p.Gln451Arg)	---	---	Uncertain significance	−0.80	1.374e-05	15.79
Insufficient	I1	*CYP4F22*	NM_173483.3	c.160C>T (p.Arg54Cys)	Ichthyosis, congenital, autosomal recessive 5	Uncertain significance	Uncertain significance	−0.37	0.0004	21.0
	I2	*CELSR1*	NM_014246.3	c.7313G>A (p.Arg2438Gln)	---	---	Benign	−2.82	0.0005	16.37
	I3	*CELSR3*	NM_001407.2	c.5875G>A (p.Ala1959Thr)	---	---	Likely Benign	−4.27	5.897e-05	23.2
	I4	*CELSR3*	NM_001407.2	c.9098G>A (p.Arg3033His)	---	---	Benign	−4.26	0.0004	24.7
	I5	*CELSR3*	NM_001407.2	c.9566G>A (p.Arg3189Gln)	---	---	Benign	−4.27	0.0008	19.39
		*TP63*	NM_001329148.1	c.1518G>T (p.Met506Ile)	ADULT syndrome; Ectrodactyly, ectodermal dysplasia, and cleft lip/palate syndrome 3, autosomal dominant	---	Uncertain significance	−0.91	8.238e-06	24.0
		*WNT11*	NM_004626.2	c.198G>A (p.Met66Ile)	---	---	Benign	−0.09	0.0003	25.7

## Discussion

BEEC is a rare congenital anomaly that requires multi-specialty care and surgical reconstruction. New technologies, including whole exome sequencing, are elucidating the underlying genetic etiologies of this complex condition (Petrovski et al., 2019). We identified rare single nucleotide candidate causative variants in DNA isolated from bladder tissue of patients with BEEC that may be related to the bladder regenerative capacity.

Three of our patients had a rare candidate causative variant in *CELSR3,* which encodes a cadherin epidermal growth factor-like laminin G-like seven-pass G-type receptor that is involved in polarity signaling (Fenstermaker et al., 2010). These were rare missense variants [c.5875G>A (p.Ala1959Thr), c.9098G>A (p.Arg3033His), c.9566G>A (p.Arg3189Gln)] in three unrelated patients with insufficient bladder regeneration (Table 3). Interestingly, Reutter et al. identified a patient with cloacal exstrophy, a subtype of BEEC, who was a compound heterozygote for two *CELSR3* coding variants [c.5470G>A (p.Val1824Met) and c.6950T>C (p.Met2317Tyr)] (Reutter et al., 2016). *CELSR3* is involved in the WNT pathway and *Celsr3* knockout mice exhibit perinatal lethality (Pitsava et al., 2021). *De novo* variants in *TUBE1* another gene on our BEEC gene list (Table 2) also functions in centriole positioning, thus strengthening their classification as candidate genes (Pitsava et al., 2021).

The *WIZ* variant identified in patient S1 (sufficient bladder) and the *CYP4F22* and *TP63* variants identified in patient l1 and l5, respectively (insufficient bladders) are classified as variants of uncertain significance by Varsome (Kopanos et al., 2019). *WIZ* is perhaps the most interesting candidate to evaluate for causation because homozygosity for null alleles results in embryonic lethality in mouse models (Daxinger et al., 2013).

*TP63* has been previously reported to be associated with bladder exstrophy (Qi et al., 2013) and the mouse *Tp63* knockout (Yang et al., 1999; Cheng et al., 2006) model presents with classic exstrophy of the bladder (Ching et al., 2010). Furthermore, Ching et al. (2010) documented reproducible dysregulation of variable tissue-specific *TP63* isoform expression in 11 of 15 BEEC patients without obvious coding p63 gene variants, suggesting alterations in *TP63* expression may be causative (Ching et al., 2010). These observations add support for a pathogenic role for the *TP63* variant c.1518G>T (p.Met506Ile)identified in one of our patients.

We performed whole exome sequencing of DNA isolated from bladder tissue in 12 BEEC patients from several different ancestries. We also provide vital information regarding the demographics and phenotypic information of patients with BEEC including bladder filling capacity. Our utilization of DNA isolated from the affected bladder tissue can also capture somatic mutations that could be present only in the bladder and absent in DNA from blood and saliva. Unfortunately, our access was limited to patient’s samples only, so we were not able to determine if a variant was inherited or *de novo*. For that reason, that we chose to limit analysis to rare heterozygous variants in genes that have been previously implicated in bladder exstrophy.

In conclusion, we present a unique series of BEEC cases that were categorized by bladder size and evaluated by whole exome sequencing. We identified rare variants in genes previously been proposed as BEEC candidates. Future steps will include obtaining parental samples and sequencing blood or saliva DNA samples of cases to assist in determining whether the mutations identified in this study are confined to the bladder (somatic) or germline. Additional investigation into the functional analysis of the identified variants will be necessary to determine causality.

## Data Availability

The original contributions presented in the study are publicly available. This data can be found here: ClinVar repository, accession number SUB14445977, https://www.ncbi.nlm.nih.gov/clinvar/?term=SUB14445977.
