# Supplementary material for: Rare exonic CELSR3 variants identified in Bladder Exstrophy Epispadias Complex
Source: Front Genet. 2024 Jun 5;15:1266210. doi: 10.3389/fgene.2024.1266210 (PMC11188427; doi:10.3389/fgene.2024.1266210)
Supplement: Supplementary file 1 [file DataSheet1.docx]

Appendix A. Genes implicated in BEEC identified by literature review

| **Gene** | **Name** | **Pathway/Gene set** | **OMIM** | **Mode of inheritance** | **Mice Knockout** | **Ref** |
| --- | --- | --- | --- | --- | --- | --- |
| AKAP8L | A-kinase Anchor protein 8-like protein | CTBP2 | ---- | ---- | Unknown | ^[[1]](#endnote-1)^ |
| ALX4 | Aristaless-like homeobox 4 | CTBP2 | Frontonasal dysplasia 2 | Autosomal recessive | Skull defect, craniosynostosis, hypertelorism, depressed nasal bridge, cryptorchidism, agenesis of the corpus collosum. | ^[[2]](#endnote-2)^ |
| AR | Androgen Receptor | AKT signaling pathway | Androgen insensitivity | X-linked recessive | Males have female external genitalia, hypospadias | ^[[3]](#endnote-3)^ |
| BMP10 | Bone Morphogenetic Protein 10 | TGF-beta | -- | -- | Decreased embryo size, cardiac dysgenesis, enlarged pericardium, arteriovenous malformations, and embryonic lethality | ^[[4]](#endnote-4)^ |
| BMP4 | Bone Morphogenetic Protein 4 | TGF-beta | Microphthalmia, syndromic 6 and Orofacial cleft 11 | Autosomal dominant | Pelvic/urogenital organ aplasia | ^[[5]](#endnote-5)^ |
| BRD4 | Human | BET family of nuclear proteins | --- | --- | Nephrocalcinosis | ^[[6]](#endnote-6)^ |
| CASP14 | Human | Caspase | Ichthyosis, congenital, autosomal recessive 12 | Autosomal recessive | Shiny lichenified epidermis | ^[[7]](#endnote-7)^ |
| CELSR1 | Human | WNT/PCP | --- | --- | Neural tube defects, abnormal hair follicle orientation, whorl-like hair patterns, and partial prenatal lethality. | ^[[8]](#endnote-8)^ |
| CELSR3 | Human | WNT/PCP | --- | --- | Neonatal lethality, abnormal nervous system development, and abnormal respiratory system development. | ^[[9]](#endnote-9)^ |
| CRKL | Human | Protooncogene | --- | --- | Abnormal neural crest derivatives including cranial ganglia, aortic arch arteries, cardiac outflow tract, thymus and parathyroid glands. | ^[[10]](#endnote-10),^^[[11]](#endnote-11)^. |
| CYP4F22 | Human | Cytochrome P450 | Ichthyosis, congenital, autosomal recessive 5 | Autosomal recessive | Impaired epidermis, neonatal demise^[[12]](#endnote-12)^ | ^[[13]](#endnote-13)^  ^[[14]](#endnote-14)^ |
| DVL1 | Human | WNT pathway | Robinow syndrome | Autosomal dominant | Reduced social interaction, decreased whisker trimming, deficits in nest building, subordinate response | ^[[15]](#endnote-15)^ |
| DKK1 | Human | WNT pathway | --- | --- | Neonatal demise with morphologic defects including a lack of anterior head structures and duplications and fusions of limb digits | ^[[16]](#endnote-16)^ |
| EFNB1 | Human | Eph family | Craniofrontonasal syndrome | X-linked dominant | Partial perinatal lethality, abdominal wall closure defects and skeletal abnormalities | ^[[17]](#endnote-17)^,^[[18]](#endnote-18)^ |
| FGF8 | Human | Growth factor | Hypogonadotropic hypogonadism 6 with or without anosmia | Autosomal dominant | Brain malformation, abnormal development of the hear, posterior compartment, or craniofacial Meyers EN, Lewandoski M, Martin GR. An Fgf8 mutant allelic series generated by Cre- and Flp-mediated recombination. *Nat Genet*. 1998;18(2):136-141. doi:10.1038/ng0298-136 structures. | ^[[19]](#endnote-19)^ |
| GLI3 | Human | Hedgehog signaling pathway | Greig cephalopolysyndactyly syndrome | Autosomal Dominant | Homozygosity for the null mutation is embryonic lethal due to skeletal defects | ^[[20]](#endnote-20)^ |
| IHH | Human | Hedgehog signaling pathway | Acrocapitofemoral dysplasia | Autosomal recessive | Abnormal long bones | ^[[21]](#endnote-21)^  ^[[22]](#endnote-22)^ |
| ISL1 | Human | LIM/homeobox | --- | --- | Embryonic demise, abnormal hearts^[[23]](#endnote-23)^ | ^[[24]](#endnote-24)^  ^[[25]](#endnote-25)^ |
| LRP6 | Human | Low density lipoprotein receptor gene family (LDLR) | Tooth agenesis, selective, 7 | Autosomal dominant | Vertebral malformation, neural tube defects, abnormal somite segmentation^[[26]](#endnote-26)^ | ^[[27]](#endnote-27)^ |
| LZTR1 | Human | RAS-MAPK signaling | Noonan syndrome 2 and 10 | Autosomal recessive, autosomal dominant | Embryonic lethal, cardiac malformations^[[28]](#endnote-28)^ | ^[[29]](#endnote-29)^ ^[[30]](#endnote-30)^  ^[[31]](#endnote-31)^ |
| MAMLD1 | Human | Chromosome X open reading frame 6 (CXORF6) | Hypospadias 2, X-linked | X-linked recessive | -- | ^[[32]](#endnote-32)^ |
| MSX1 | Human | HOX genes | Ectodermal dysplasia 3, Witkop type; Orofacial cleft 5; Tooth agenesis, selective, 1, with or without orofacial cleft | Autosomal dominant | Neonatal demise, craniofacial defects including cleft palate, reduced mandible and maxilla, and retarded tooth development. | ^[[33]](#endnote-33)^ |
| NOC2L | Human | Histone deacetylases (HDAC) | --- | --- | --- | ^[[34]](#endnote-34)^ |
| OTX1 | Human | Homeobox | --- | --- | Epilepsy, abnormal brains | ^[[35]](#endnote-35)^ |
| p63 |  | Tumor protein | ADULT syndrome, Hay-Wells syndrome, Limb-mammary syndreom | Autosomal dominant | Defects in limb, craniofacial, and epithelial development | ^[[36]](#endnote-36)^  **^[[37]](#endnote-37)^** |
| PITX1 | Human | Homeobox | Clubfoot, congenital, with or without deficiency of long bones and/or mirror-image polydactyly | Autosomal dominant | Hindlimb abnormality, branchial arch abnormality, cleft palate | ^[[38]](#endnote-38)^ |
| PLAG1 |  | Zinc Finger | Adenomas, salivary gland pleomorphic  Silver-Russell syndrome 4 | Autosomal dominant | Reduced fertility^[[39]](#endnote-39)^ | ^[[40]](#endnote-40)^ |
| SHH | Human | Hedgehog signaling pathway | Holoprosencephaly 3, micropthalmia with coloboma 5 | Autosomal Dominant | Midline defects, defects in distal limbs, cyclopia, neural tube, and ribs^[[41]](#endnote-41)^ | ^[[42]](#endnote-42)^ |
| SHOX | Human | Homeobox | Langer mesomelic dysplasia, Leri-Well dyschondrosteosis | Pseudoautosomal recessive, pseudoautosomal dominant | Markedly shortened limbs^[[43]](#endnote-43)^ | ^[[44]](#endnote-44)^ |
| SLC20A1 | Human | Phosphate transporter | --- | --- | Embryonic lethal | ^[[45]](#endnote-45)^ |
| SYDE1 | Human | GTPase Homolog | --- | --- | Normal and fertile | ^[[46]](#endnote-46)^ |
| TGF-B | Human | TGF-beta | Camurati-Engelmann disease | Autosomal Dominant | Poor wound healing, fibrosis^[[47]](#endnote-47)^ | ^[[48]](#endnote-48)^ |
| THAP7 | Human | THAP Domain | --- | --- | --- | ^[[49]](#endnote-49)^ |
| TP63 | Human | Tumor Protein | ADULT syndrome; Ectrodactyly, ectodermal dysplasia, and cleft lip/palate syndrome 3; Hay-Wells syndrome; Limb-mammary syndrome; Orofacial cleft 8; Rapp-Hodgkin syndrome; Split-hand/foot malformation 4 | Autosomal dominant | Prenatal demise, craniofacial anomalies, missing or truncated limbs, and small genitalia. | ^[[50]](#endnote-50)^ |
| UPB1 | Human | Pyrimidine degradation | Beta-ureidopropionase deficiency | Autosomal recessive | --- | ^[[51]](#endnote-51)^Yaplito-Lee, J., Pitt, J., Meijer, J., Zoetekouw, L., Meinsma, R., van Kuilenburg, A. B. P. **Beta-ureidopropionase deficiency presenting with congenital anomalies of the urogenital and colorectal systems.** Molec. Genet. Metab. 93: 190-194, 2008 Yaplito-Lee, J., Pitt, J., Meijer, J., Zoetekouw, L., Meinsma, R., van Kuilenburg, A. B. P. **Beta-ureidopropionase deficiency presenting with congenital anomalies of the urogenital and colorectal systems.** Molec. Genet. Metab. 93: 190-194, 2008 |
| WIZ | Human | Zinc Finger | --- | --- | Altered behavior | ^[[52]](#endnote-52)^ |
| WNT10A | Human | WNT/PCP | Odontoonychodermal dysplasia; Schopf-Schulz-Passarge syndrome; Tooth agenesis, selective, 4 | Autosomal dominant, Autosomal recessive | Taurodontism, supernumerary molars, small molars and misshaped crowns | ^[[53]](#endnote-53)^ |
| WNT11 | Human | WNT/PCP | --- | --- | Perinatal lethality, kidneys are small and exhibit delayed development | ^[[54]](#endnote-54)^ |
| WNT2B | Human | WNT/PCP | Diarrhea 9 | Autosomal recessive | --- | ^[[55]](#endnote-55)^ |
| WNT3 | Human | WNT/PCP | Tetra-amelia syndrome 1 | Autosomal recessive | Embryologic demise | ^[[56]](#endnote-56),^^[[57]](#endnote-57)^ |
| WNT5A | Human | WNT/PCP | Robinow syndrome, autosomal dominant 1 | Autosomal dominant | Dwarfism, facial abnormalities, short limbs, dysplasia of lungs and genitals, ventricular septal defects | ^[[58]](#endnote-58)^ |
| WNT6 | Human | WNT/PCP | --- | --- | Impaired decidualization with reduced uterine stromal cells | ^[[59]](#endnote-59)^ |
| WNT9b | Human | WNT/PCP | --- | --- | Neonatal demise, vestigial kidneys and lack of reproductive ducts | ^[[60]](#endnote-60),^^[[61]](#endnote-61)^ |
| WNT11 | Human | WNT/PCP | --- | --- | Cardiac defects | ^[[62]](#endnote-62)^ |

1. Draaken M, Mughal SS, Pennimpede T, Wolter S, Wittler L, Ebert AK, Rösch W, Stein R, Bartels E, Schmidt D, Boemers TM, Schmiedeke E, Hoffmann P, Moebus S, Herrmann BG, Nöthen MM, Reutter H, Ludwig M. Isolated bladder exstrophy associated with a de novo 0.9 Mb microduplication on chromosome 19p13.12. Birth Defects Res A Clin Mol Teratol. 2013 Mar; 97(3):133-9. [↑](#endnote-ref-1)
2. Matsumaru D, Haraguchi R, Moon AM, et al. Genetic analysis of the role of Alx4 in the coordination of lower body and external genitalia formation. Eur J Hum Genet. 2014;22(3):350-357. doi:10.1038/ejhg.2013.160 [↑](#endnote-ref-2)
3. Batch, J. A., Evans, B. A. J., Hughes, I. A., Patterson, M. N. **Mutations of the androgen receptor gene identified in perineal hypospadias.** J. Med. Genet. 30: 198-201, 1993 [↑](#endnote-ref-3)
4. Suzuki K, Adachi Y, Numata T, Nakada S, Yanagita M, Nakagata N, Evans SM, Graf D, Economides A, Haraguchi R, Moon AM, Yamada G Reduced BMP signaling results in hindlimb fusion with lethal pelvic/urogenital organ aplasia: a new mouse model of sirenomelia. PLoS One. 2012; 7(9):e43453. [↑](#endnote-ref-4)
5. Ching ST, Infante CR, Du W, Sharir A, Park S, Menke DB, Klein OD. Isl1 mediates mesenchymal expansion in the developing external genitalia via regulation of Bmp4, Fgf10 and Wnt5a. Hum Mol Genet. 2018 Jan 1;27(1):107-119. doi: 10.1093/hmg/ddx388. PMID: 29126155; PMCID: PMC5886284. [↑](#endnote-ref-5)
6. Draaken M, Mughal SS, Pennimpede T, Wolter S, Wittler L, Ebert AK, Rösch W, Stein R, Bartels E, Schmidt D, Boemers TM, Schmiedeke E, Hoffmann P, Moebus S, Herrmann BG, Nöthen MM, Reutter H, Ludwig M. Isolated bladder exstrophy associated with a de novo 0.9 Mb microduplication on chromosome 19p13.12. Birth Defects Res A Clin Mol Teratol. 2013 Mar; 97(3):133-9. [↑](#endnote-ref-6)
7. Draaken M, Mughal SS, Pennimpede T, Wolter S, Wittler L, Ebert AK, Rösch W, Stein R, Bartels E, Schmidt D, Boemers TM, Schmiedeke E, Hoffmann P, Moebus S, Herrmann BG, Nöthen MM, Reutter H, Ludwig M. Isolated bladder exstrophy associated with a de novo 0.9 Mb microduplication on chromosome 19p13.12. Birth Defects Res A Clin Mol Teratol. 2013 Mar; 97(3):133-9. [↑](#endnote-ref-7)
8. Curtin JA, Quint E, Tsipouri V, et al. Mutation of Celsr1 disrupts planar polarity of inner ear hair cells and causes severe neural tube defects in the mouse. Curr Biol. 2003;13(13):1129-1133. doi:10.1016/s0960-9822(03)00374-9 [↑](#endnote-ref-8)
9. Reutter H, Keppler-Noreuil K, E Keegan C, Thiele H, Yamada G, Ludwig M. Genetics of Bladder-Exstrophy-Epispadias Complex (BEEC): Systematic Elucidation of Mendelian and Multifactorial Phenotypes. Curr Genomics. 2016;17(1):4-13. doi:10.2174/1389202916666151014221806 [↑](#endnote-ref-9)
10. Guris DL, Fantes J, Tara D, Druker BJ, Imamoto A. Mice lacking the homologue of the human 22q11.2 gene CRKL phenocopy neurocristopathies of DiGeorge syndrome. Nat Genet. 2001;27(3):293-298. doi:10.1038/85855 [↑](#endnote-ref-10)
11. Draaken M, Baudisch F, Timmermann B, Kuhl H, Kerick M, Proske J, Wittler L, Pennimpede T, Ebert AK, Rösch W, Stein R, Bartels E, von Lowtzow C, Boemers TM, Herms S, Gearhart JP, Lakshmanan Y, Kockum CC, Holmdahl G, Läckgren G, Nordenskjöld A, Boyadjiev SA, Herrmann BG, Nöthen MM, Ludwig M, Reutter H. Classic bladder exstrophy: Frequent 22q11.21 duplications and definition of a 414 kb phenocritical region. Birth Defects Res A Clin Mol Teratol. 2014 Jun; 100(6):512-7. [↑](#endnote-ref-11)
12. Miyamoto M, Itoh N, Sawai M, Sassa T, Kihara A. Severe Skin Permeability BarrierDysfunction in Knockout Mice Deficient in a Fatty Acid ω-Hydroxylase Crucial to Acylceramide Production. J Invest Dermatol. 2020 Feb;140(2):319-326.e4. doi: 10.1016/j.jid.2019.07.689. Epub 2019 Jul 26. PMID: 31356814. [↑](#endnote-ref-12)
13. Lefèvre C, Bouadjar B, Ferrand V, et al. Mutations in a new cytochrome P450 gene in lamellar ichthyosis type 3. Hum Mol Genet. 2006;15(5):767-776. doi:10.1093/hmg/ddi491 [↑](#endnote-ref-13)
14. Draaken M, Mughal SS, Pennimpede T, Wolter S, Wittler L, Ebert AK, Rösch W, Stein R, Bartels E, Schmidt D, Boemers TM, Schmiedeke E, Hoffmann P, Moebus S, Herrmann BG, Nöthen MM, Reutter H, Ludwig M. Isolated bladder exstrophy associated with a de novo 0.9 Mb microduplication on chromosome 19p13.12. Birth Defects Res A Clin Mol Teratol. 2013 Mar; 97(3):133-9. [↑](#endnote-ref-14)
15. Rosso SB, Sussman D, Wynshaw-Boris A, Salinas PC. Wnt signaling through Dishevelled, Rac and JNK regulates dendritic development. Nat Neurosci. 2005 Jan; 8(1):34-42 [↑](#endnote-ref-15)
16. Mukhopadhyay, M., Shtrom, S., Rodriguez-Esteban, C., Chen, L., Tsukui, T., Gomer, L., Dorward, D. W., Glinka, A., Grinberg, A., Huang, S.-P., Niehrs, C., Belmonte, J. C. I., Westphal, H. **Dickkopf1 is required for embryonic head induction and limb morphogenesis in the mouse.** Dev. Cell 1: 423-434, 2001 [↑](#endnote-ref-16)
17. Ludwig M, Ching B, Reutter H, Boyadjiev SA. Bladder exstrophy-epispadias complex. Birth Defects Res Part A Clin Mol Teratol. 2009;85(6):509–22 [↑](#endnote-ref-17)
18. Compagni A, Logan M, Klein R, Adams RH. Control of skeletal patterning by ephrinB1-EphB interactions.Dev. Cell 5:217-230, 2003’ [↑](#endnote-ref-18)
19. Comprehensive genetic analysis of OEIS complex reveals no evidence for a recurrent microdeletion or duplication.

    Vlangos CN, Siuniak A, Ackley T, van Bokhoven H, Veltman J, Iyer R, Park JM, Keppler-Noreuil K, Keegan CE

    Am J Med Genet A. 2011 Jan; 155A(1):38-49. [↑](#endnote-ref-19)
20. Matera, I., Watkins-Chow, D. E., Loftus, S. K., Hou, L., Incao, A., Silver, D. L., Rivas, C., Elliott, E. C., Baxter, L. L., Pavan, W. J. **A sensitized mutagenesis screen identifies Gli3 as a modifier of Sox10 neurocristopathy.** Hum. Molec. Genet. 17: 2118-2131, 2008.

    Genetic analysis of the role of Alx4 in the coordination of lower body and external genitalia formation.

    Matsumaru D, Haraguchi R, Moon AM, Satoh Y, Nakagata N, Yamamura K, Takahashi N, Kitazawa S, Yamada G

    Eur J Hum Genet. 2014 Mar; 22(3):350-7. [↑](#endnote-ref-20)
21. Maeda, Y., Nakamura, E., Nguyen, M.-T., Suva, L. J., Swain, F. L., Razzaque, M. S., Mackem, S., Lanske, B. **Indian hedgehog produced by postnatal chondrocytes is essential for maintaining a growth plate and trabecular bone.** Proc. Nat. Acad. Sci. 104: 6382-6387, 2007. [↑](#endnote-ref-21)
22. Comprehensive genetic analysis of OEIS complex reveals no evidence for a recurrent microdeletion or duplication.

    Vlangos CN, Siuniak A, Ackley T, van Bokhoven H, Veltman J, Iyer R, Park JM, Keppler-Noreuil K, Keegan CE

    Am J Med Genet A. 2011 Jan; 155A(1):38-49. [↑](#endnote-ref-22)
23. Cai CL, Liang X, Shi Y, et al. Isl1 identifies a cardiac progenitor population that proliferates prior to differentiation and contributes a majority of cells to the heart. Dev Cell. 2003;5(6):877-889. doi:10.1016/s1534-5807(03)00363-0. [↑](#endnote-ref-23)
24. CHING

    Draaken M, Knapp M,

    Pennimpede T, et al. (2015)  Genome-wide association study and meta-analysis identify ISL1 as genome-wide significant susceptibility gene for bladder exstrophy. PLoS Genet.

    , 11, e1005024 [↑](#endnote-ref-24)
25. Sharma A, Dakal TC, Ludwig M, Fröhlich H, Mathur R, Reutter H. Towards a Central Role of ISL1 in the Bladder Exstrophy⁻Epispadias Complex (BEEC): Computational Characterization of Genetic Variants and Structural Modelling. Genes (Basel). 2018 Dec 5;9(12):609. doi: 10.3390/genes9120609. PMID: 30563179; PMCID: PMC6315746. [↑](#endnote-ref-25)
26. Kokubu C, Heinzmann U, Kokubu T, et al. Skeletal defects in ringelschwanz mutant mice reveal that Lrp6 is required for proper somitogenesis and osteogenesis. Development. 2004;131(21):5469-5480. doi:10.1242/dev.01405 [↑](#endnote-ref-26)
27. Wnt induces LRP6 signalosomes and promotes dishevelled-dependent LRP6 phosphorylation.

    Bilic J, Huang YL, Davidson G, Zimmermann T, Cruciat CM, Bienz M, Niehrs C

    Science. 2007 Jun 15; 316(5831):1619-22. [↑](#endnote-ref-27)
28. Piotrowski A, Xie J, Liu YF, et al. Germline loss-of-function mutations in LZTR1 predispose to an inherited disorder of multiple schwannomas. Nat Genet. 2014;46(2):182-187. doi:10.1038/ng.2855 [↑](#endnote-ref-28)
29. Pierquin G., Uwineza A. 22q11.2 microduplication in a patient with bladder exstrophy and delayed psychomotor development. Eur. J. Hum. Genet. 2012;20(Suppl. 1):89. [abstr]. [↑](#endnote-ref-29)
30. Lundin J, Markljung E, Baranowska Körberg I, Hofmeister W, Cao J, Nilsson D, Holmdahl G, Barker G, Anderberg M, Vukojević V, Lindstrand A, Nordenskjöld A. Further support linking the 22q11.2 microduplication to an increased risk of bladder exstrophy and highlighting LZTR1 as a candidate gene. Mol Genet Genomic Med. 2019 Jun;7(6):e666. doi: 10.1002/mgg3.666. Epub 2019 May 1. PMID: 31044557; PMCID: PMC6565582. [↑](#endnote-ref-30)
31. Classic bladder exstrophy: Frequent 22q11.21 duplications and definition of a 414 kb phenocritical region.

    Draaken M, Baudisch F, Timmermann B, Kuhl H, Kerick M, Proske J, Wittler L, Pennimpede T, Ebert AK, Rösch W, Stein R, Bartels E, von Lowtzow C, Boemers TM, Herms S, Gearhart JP, Lakshmanan Y, Kockum CC, Holmdahl G, Läckgren G, Nordenskjöld A, Boyadjiev SA, Herrmann BG, Nöthen MM, Ludwig M, Reutter H. Teratol. 2014 Jun; 100(6):512-7.

    Birth Defects Res A Clin Mol [↑](#endnote-ref-31)
32. Fukami, M., Wada, Y., Miyabayashi, K. et al. CXorf6 is a causative gene for hypospadias. Nat Genet 38, 1369–1371 (2006). https://doi.org/10.1038/ng1900 [↑](#endnote-ref-32)
33. Jumlongras D, Bei M, Stimson JM, et al. A nonsense mutation in MSX1 causes Witkop syndrome. Am J Hum Genet. 2001;69(1):67-74. doi:10.1086/321271 [↑](#endnote-ref-33)
34. Wnt signaling through Dishevelled, Rac and JNK regulates dendritic development.

    Rosso SB, Sussman D, Wynshaw-Boris A, Salinas PC

    Nat Neurosci. 2005 Jan; 8(1):34-42 [↑](#endnote-ref-34)
35. Acampora D, Mazan S, Avantaggiato V, et al. Epilepsy and brain abnormalities in mice lacking the Otx1 gene. Nat Genet. 1996;14(2):218-222. doi:10.1038/ng1096-218 [↑](#endnote-ref-35)
36. Mahfuz I, Darling T, Wilkins S, White S, Cheng W. New insights into the pathogenesis of bladder exstrophy-epispadias complex. J Pediatr Urol. 2013;9(6 Pt B):996-1005. doi:10.1016/j.jpurol.2013.05.001 [↑](#endnote-ref-36)
37. Yang, A., Schweitzer, R., Sun, D., Kaghad, M., Walker, N., Bronson, R. T., Tabin, C., Sharpe, A., Caput, D., Crum, C., McKeon, F. **p63 is essential for regenerative proliferation in limb, craniofacial and epithelial development** Nature 398: 714-718, 1999. [↑](#endnote-ref-37)
38. Szeto DP, Rodriguez-Esteban C, Ryan AK, et al. Role of the Bicoid-related homeodomain factor Pitx1 in specifying hindlimb morphogenesis and pituitary development. Genes Dev. 1999;13(4):484-494. doi:10.1101/gad.13.4.484 [↑](#endnote-ref-38)
39. Karim, L., Takeda, H., Lin, L., Druet, T., Arias, J. A. C., Baurain, D., Cambisano, N., Davis, S. R., Farnir, F., Grisart, B., Harris, B. L., Keehan, M. D., Littlejohn, M. D., Spelman, R. J., Georges, M., Coppieters, W. **Variants modulating the expression of a chromosome domain encompassing PLAG1 influence bovine stature.** Nature Genet. 43: 405-413, 2011 [↑](#endnote-ref-39)
40. Kolarova J, Bens S, Ammerpohl O, Hilger AC, Zhang R, Reutter H, Siebert R. PLAGL1 epimutation and bladder exstrophy: Coincidence or concurrent etiology? Birth Defects Res A Clin Mol Teratol. 2016 Aug;106(8):724-8. doi: 10.1002/bdra.23521. Epub 2016 May 25. PMID: 27223093 [↑](#endnote-ref-40)
41. Chiang, C., Litingtung, Y., Lee, E., Young, K. E., Corden, J. L., Westphal, H., Beachy, P. A. **Cyclopia and defective axial patterning in mice lacking Sonic hedgehog gene function.** Nature 383: 407-413, 1996 [↑](#endnote-ref-41)
42. Genetic analysis of Hedgehog signaling in ventral body wall development and the onset of omphalocele formation.

    Matsumaru D, Haraguchi R, Miyagawa S, Motoyama J, Nakagata N, Meijlink F, Yamada G

    PLoS One. 2011 Jan 20; 6(1):e16260. [↑](#endnote-ref-42)
43. Cobb J, Dierich A, Huss-Garcia Y, Duboule D. A mouse model for human short-stature syndromes identifies Shox2 as an upstream regulator of Runx2 during long-bone development. Proc Natl Acad Sci U S A. 2006;103(12):4511-4515. doi:10.1073/pnas.0510544103 [↑](#endnote-ref-43)
44. A case with bladder exstrophy and unbalanced X chromosome rearrangement.

    Soderhall C, Lundin J, Lagerstedt-Robinson K, Grigelioniene G, Lackgren G, Kockum CC, Nordenskjold A

    Eur J Pediatr Surg. 2014 Aug; 24(4):353-9. [↑](#endnote-ref-44)
45. Rieke JM, Zhang R, Braun D, et al. SLC20A1 Is Involved in Urinary Tract and Urorectal Development. Front Cell Dev Biol. 2020 Aug 7;8:567. doi: 10.3389/fcell.2020.00567. PMID: 32850778; PMCID: PMC7426641. [↑](#endnote-ref-45)
46. Isolated bladder exstrophy associated with a de novo 0.9 Mb microduplication on chromosome 19p13.12.

    Draaken M, Mughal SS, Pennimpede T, Wolter S, Wittler L, Ebert AK, Rösch W, Stein R, Bartels E, Schmidt D, Boemers TM, Schmiedeke E, Hoffmann P, Moebus S, Herrmann BG, Nöthen MM, Reutter H, Ludwig M

    Birth Defects Res A Clin Mol Teratol. 2013 Mar; 97(3):133-9. [↑](#endnote-ref-46)
47. Clouthier, D. E., Comerford, S. A., Hammer, R. E. **Hepatic fibrosis, glomerulosclerosis, and lipodystrophy-like PEPCK-TGF-beta-1 transgenic mice.** J. Clin. Invest. 100: 2697-2713, 1997. [↑](#endnote-ref-47)
48. Suson KD, Stec AA, Gearhart JP, Shimoda LA. Transforming growth factor-β1 mediates migration in cultured human control and exstrophy bladder smooth muscle cells. J Urol. 2012 Oct;188(4 Suppl):1528-33. doi: 10.1016/j.juro.2012.02.038. Epub 2012 Aug 19. PMID: 22910259. [↑](#endnote-ref-48)
49. Classic bladder exstrophy: Frequent 22q11.21 duplications and definition of a 414 kb phenocritical region.

    Draaken M, Baudisch F, Timmermann B, Kuhl H, Kerick M, Proske J, Wittler L, Pennimpede T, Ebert AK, Rösch W, Stein R, Bartels E, von Lowtzow C, Boemers TM, Herms S, Gearhart JP, Lakshmanan Y, Kockum CC, Holmdahl G, Läckgren G, Nordenskjöld A, Boyadjiev SA, Herrmann BG, Nöthen MM, Ludwig M, Reutter H

    Birth Defects Res A Clin Mol Teratol. 2014 Jun; 100(6):512-7. [↑](#endnote-ref-49)
50. DeltaNp63 plays an anti-apoptotic role in ventral bladder development.

    Cheng W, Jacobs WB, Zhang JJ, Moro A, Park JH, Kushida M, Qiu W, Mills AA, Kim PC

    Development. 2006 Dec; 133(23):4783-92. Interaction between the TP63 and SHH pathways is an important determinant of epidermal homeostasis.

    Chari NS, Romano RA, Koster MI, Jaks V, Roop D, Flores ER, Teglund S, Sinha S, Gruber W, Aberger F, Medeiros LJ, Toftgard R, McDonnell TJ

    Cell Death Differ. 2013 Aug; 20(8):1080-8. [↑](#endnote-ref-50)
51. Yaplito-Lee J, Pitt J, Meijer J, Zoetekouw L, Meinsma R, van Kuilenburg AB. Beta-ureidopropionase deficiency presenting with congenital anomalies of the urogenital and colorectal systems. Mol Genet Metab. 2008;93(2):190-194. doi:10.1016/j.ymgme.2007.09.009 [↑](#endnote-ref-51)
52. Isolated bladder exstrophy associated with a de novo 0.9 Mb microduplication on chromosome 19p13.12.

    Draaken M, Mughal SS, Pennimpede T, Wolter S, Wittler L, Ebert AK, Rösch W, Stein R, Bartels E, Schmidt D, Boemers TM, Schmiedeke E, Hoffmann P, Moebus S, Herrmann BG, Nöthen MM, Reutter H, Ludwig M

    Birth Defects Res A Clin Mol Teratol. 2013 Mar; 97(3):133-9. [↑](#endnote-ref-52)
53. Yang, J., Wang, S.-K., Choi, M., Reid, B. M., Hu, Y., Lee, T.-L., Herzog, C. R., Kim-Berman, H., Lee, M., Benke, P. J., Llyod, K. C. K., Simmer, J. P., Hu, J. C.-C. **Taurodontism, variations in tooth number, and misshapened crowns in Wnt10a null mice and human kindreds.** Molec. Genet. Genomic Med. 3: 40-58, 2015. [↑](#endnote-ref-53)
54. Baranowska Körberg I, Hofmeister W, Markljung E, Cao J, Nilsson D, Ludwig M, Draaken M, Holmdahl G, Barker G, Reutter H, Vukojević V, Clementson Kockum C, Lundin J, Lindstrand A, Nordenskjöld A. WNT3 involvement in human bladder exstrophy and cloaca development in zebrafish. Hum Mol Genet. 2015 Sep 15;24(18):5069-78. doi: 10.1093/hmg/ddv225. Epub 2015 Jun 23. PMID: 26105184. [↑](#endnote-ref-54)
55. Baranowska Körberg I, Hofmeister W, Markljung E, Cao J, Nilsson D, Ludwig M, Draaken M, Holmdahl G, Barker G, Reutter H, Vukojević V, Clementson Kockum C, Lundin J, Lindstrand A, Nordenskjöld A. WNT3 involvement in human bladder exstrophy and cloaca development in zebrafish. Hum Mol Genet. 2015 Sep 15;24(18):5069-78. doi: 10.1093/hmg/ddv225. Epub 2015 Jun 23. PMID: 26105184. [↑](#endnote-ref-55)
56. Baranowska Körberg I, Hofmeister W, Markljung E, et al. WNT3 involvement in human bladder exstrophy and cloaca development in zebrafish. Hum Mol Genet. 2015;24(18):5069-5078. doi:10.1093/hmg/ddv225 [↑](#endnote-ref-56)
57. Homozygous WNT3 mutation causes tetra-amelia in a large consanguineous family.

    Niemann S, Zhao C, Pascu F, Stahl U, Aulepp U, Niswander L, Weber JL, Müller U

    Am J Hum Genet. 2004 Mar; 74(3):558-63. [↑](#endnote-ref-57)
58. Oishi, I., Suzuki, H., Onishi, N., Takada, R., Kani, S., Ohkawara, B., Koshida, I., Suzuki, K., Yamada, G., Schwabe, G. C., Mundlos, S., Shibuya, H., Takada, S., Minami, Y. **The receptor tyrosine kinase Ror2 is involved in non-canonical Wnt5a/JNK signalling pathway.** Genes Cells 8: 645-654, 2003. [↑](#endnote-ref-58)
59. Katoh M. WNT and FGF gene clusters (review). Int J Oncol. 2002 Dec;21(6):1269-73. PMID: 12429977. [↑](#endnote-ref-59)
60. Carroll, T. J., Park, J.-S., Hayashi, S., Majumdar, A., McMahon, A. P. **Wnt9b plays a central role in the regulation of mesenchymal to epithelial transitions underlying organogenesis of the mammalian urogenital system.** Dev. Cell 9: 283-292, 2005 [↑](#endnote-ref-60)
61. Reutter H, Draaken M, Pennimpede T et al. Genome-wide association

    study and mouse expression data identify a highly conserved 32 kb

    intergenic region between WNT3 and WNT9b as possible susceptibility

    locus for isolated classic exstrophy of the bladder. Hum Mol Genet 2014:

    23: 5536–5544 [↑](#endnote-ref-61)
62. Zhou, W., Lin, L., Majumdar, A., Li, X., Zhang, X., Liu, W., Etheridge, L., Shi, Y., Martin, J., Van de Ven, W., Kaartinen, V., Wynshaw-Boris, A., McMahon, A. P., Rosenfeld, M. G., Evans, S. M. **Modulation of morphogenesis by noncanonical Wnt signaling requires ATF/CREB family-mediated transcriptional activation of TGF-beta-2.** Nature Genet. 39: 1225-1234, 2007 [↑](#endnote-ref-62)
